# Supplementary material for: Urtica dioica Aqueous Leaf Extract: Chemical Composition and In Vitro Evaluation of Biological Activities
Source: Int J Mol Sci. 2025 Jan 30;26(3):1220. doi: 10.3390/ijms26031220 (PMC11818644; doi:10.3390/ijms26031220)
Supplement: Supplementary file 1 [file ijms-26-01220-s001.zip › ijms-3427838-supplementary.pdf]

Supplementary material for

Urtica dioica aqueous leaf extract: chemical composition and *in vitro* evaluation of biological activities

Nouha Dakhli[1], Auxiliadora López-Jiménez[2], Casimiro Cárdenas[2], Manel Hraoui[1], Jihene Dhaouafi[1,3], Manuel Bernal[4,5], Hichem Sebai[1], Miguel Ángel Medina[4,5,6]

[1] Laboratory of Functional Physiology and Valorization of Bio-Ressources-Higher Institute of Biotechnology of Beja, B.P. 382-9000 Beja, University of Jendouba, Tunisia

[2] Research Support Central Services (SCAI) of the University of Málaga, E-29071 Málaga, Spain

[3] UMR Transfrontalière BioEcoAgro N°1158, Université Lille, INRAE, Université Liège, UPJV, YNCREA, Université Artois, Université Littoral Côte d'Opale, ICV-Institut Charles Viollette, F-59000 Lille, France.

[4] Department of Molecular Biology and Biochemistry, Faculty of Sciences, University of Málaga, Andalucía Tech, E-29071 Málaga, Spain

[5] Málaga Biomedical Research Institute and Nanomedicine Platform (IBIMA-Plataforma BIONAND), C/Severo Ochoa, 35, E-29590 Málaga, Spain

[6] Network Biomedical Research Center for Rare Diseases (CIBERER), U741, E-28029 Málaga, Spain

Compound	Molecular formula	Mass error (ppm)	Annotation MW	m/z	RT (min)	Reference ion
2-Hydroxycinnamic acid	C9 H8 O3	0,21	164,04738	165,05466	46,229	[M+H]+1

230222_8_URTICA_20ug (F2) #13830, RT=46.232 min, MS1, FTM
C9 H8 O3 as [M+H]+1

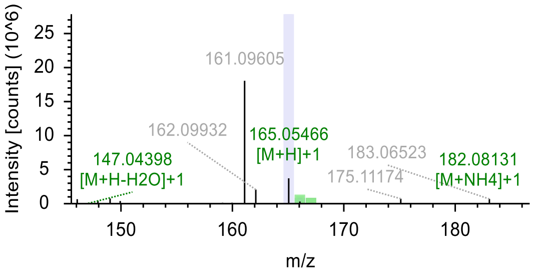

230222_8_URTICA_20ug (F2) #13837, RT=46.253 min, MS2, FTM

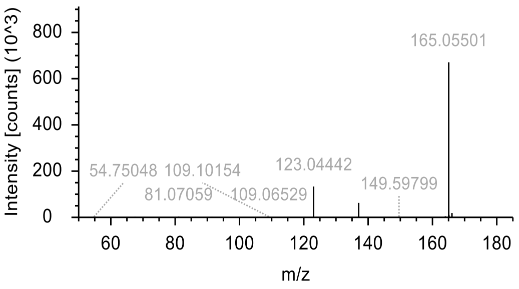

Compound	Molecular formula	Mass error (ppm)	Annotation MW	m/z	RT (min)	Reference ion
3,4-Dihydroxybenzaldehyde	C7 H6 O3	-0,33	138,03165	139,03893	45,023	[M+H]+1

230222_8_URTICA_20ug (F2) #13391, RT=45.025 min, MS1, FTM
C7 H6 O3 as [M+H]+1

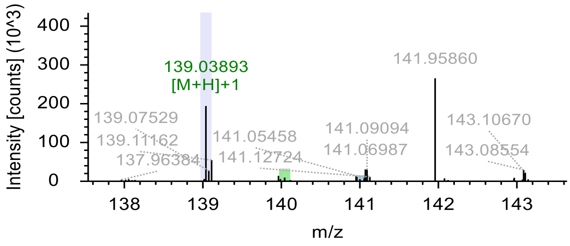

230222_8_URTICA_20ug (F2) #13393, RT=45.031 min, MS2, FTM

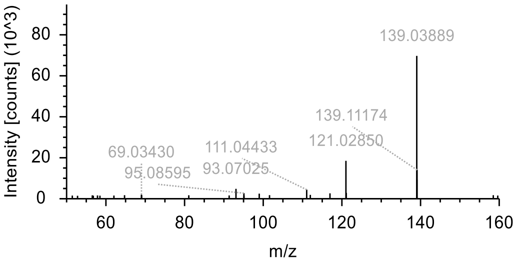

Compound	Molecular formula	Mass error (ppm)	Annotation MW	m/z	RT (min)	Reference ion
4-Hydroxycoumarine	C9 H6 O3	-0.56	162,03160	163,03888	61,499	[M+H]+1

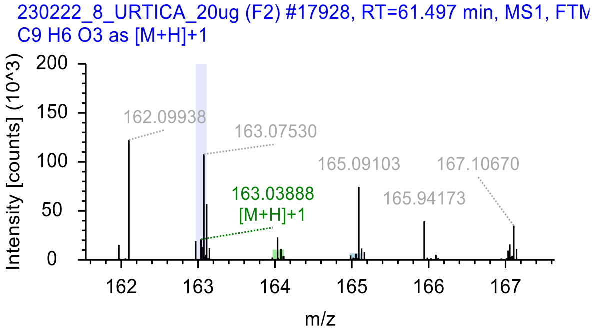

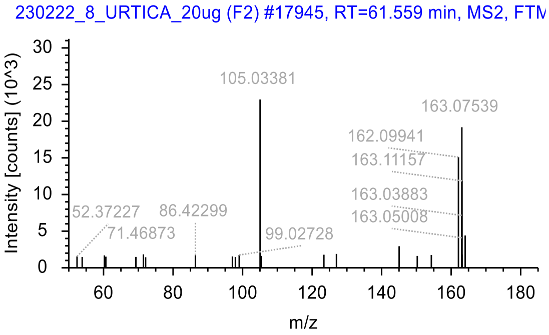

Compound	Molecular formula	Mass error (ppm)	Annotation MW	m/z	RT (min)	Reference ion
4-Methylumbelliferone hydrate	C10 H8 O3	-0,38	176,04728	209,08083	25,641	[M+H+MeOH]+1

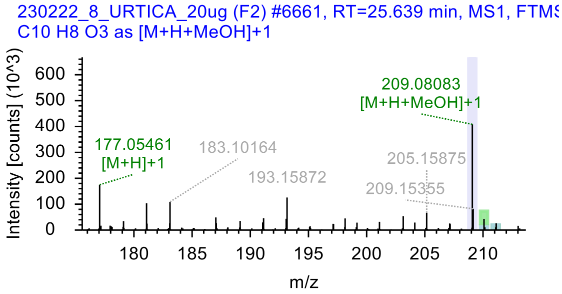

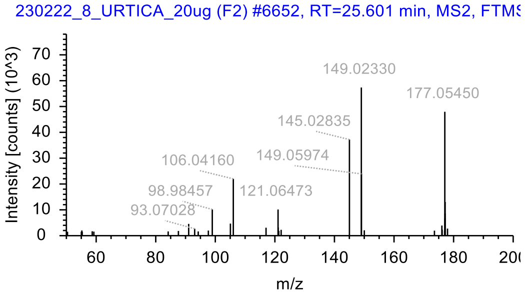

**Compound**	**Molecular formula**	**Mass error (ppm)**	**Annotation MW**	**m/z**	**RT (min)**	**Reference ion**
alpha-Bisabolol	C15 H26 O	0,35	222,19844	223,20572	47,887	[M+H]+1

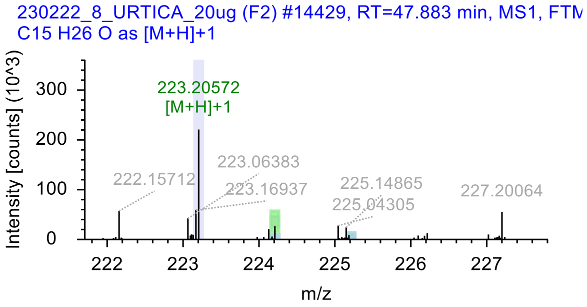

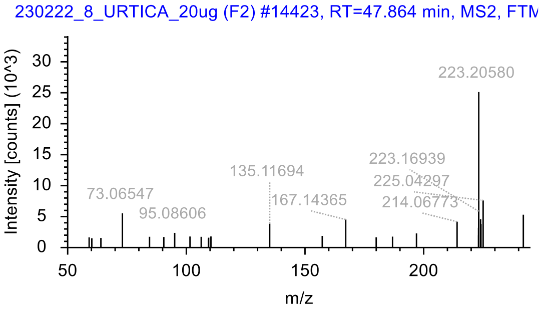

**Compound**	**Molecular formula**	**Mass error (ppm)**	**Annotation MW**	**m/z**	**RT (min)**	**Reference ion**
alpha-Bisabolol, acetate	C17 H28 O2	-0,80	264,20872	247,20554	38,134	[M+H-H2O]+1

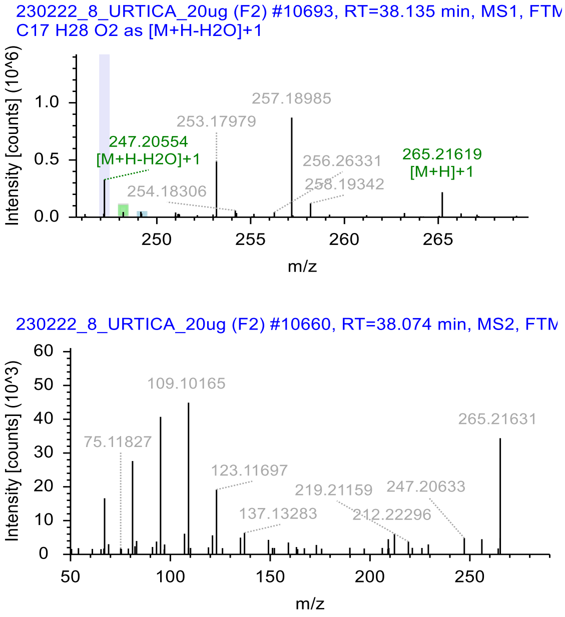

Compound	Molecular formula	Mass error (ppm)	Annotation MW	m/z	RT (min)	Reference ion
alpha-Farnesene	C15 H24	0.27	204,18786	205,19513	46,802	[M+H]+1

230222_8_URTICA_20ug (F2) #14016, RT=46.799 min, MS1, FTM
C15 H24 as [M+H]+1

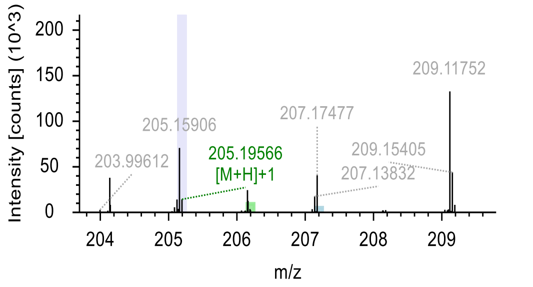

230222_8_URTICA_20ug (F2) #14068, RT=46.955 min, MS2, FTM

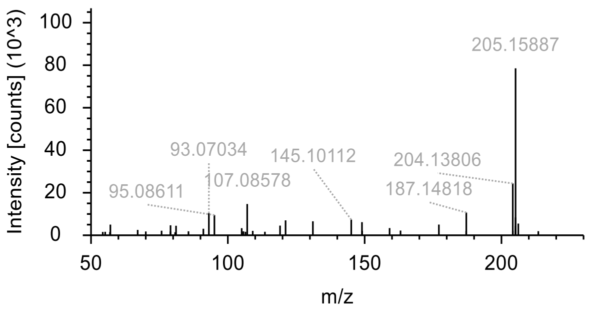

Compound	Molecular formula	Mass error (ppm)	Annotation MW	m/z	RT (min)	Reference ion
alpha-Pinene	C10 H16	1,79	136,12544	137,13272	0.094	[M+H]+1

230222_8_URTICA_20ug (F2) #39, RT=0.097 min, MS1, FTMS (+
C10 H16 as [M+H]+1

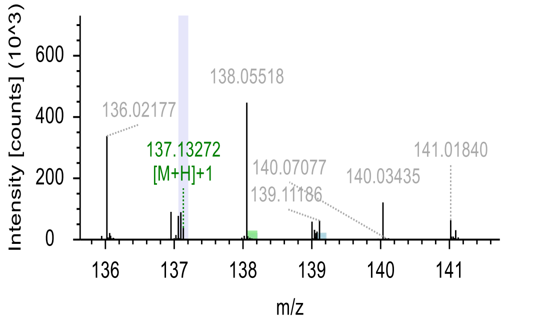

230222_8_URTICA_20ug (F2) #8, RT=0.022 min, MS2, FTMS (+),

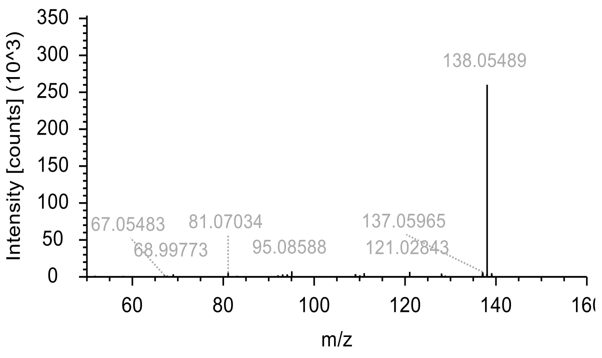

Compound	Molecular formula	Mass error (ppm)	Annotation MW	m/z	RT (min)	Reference ion

Angelic acid	C5 H8 O2	3,98	100,05283	101,06010	41,247	[M+H]+1

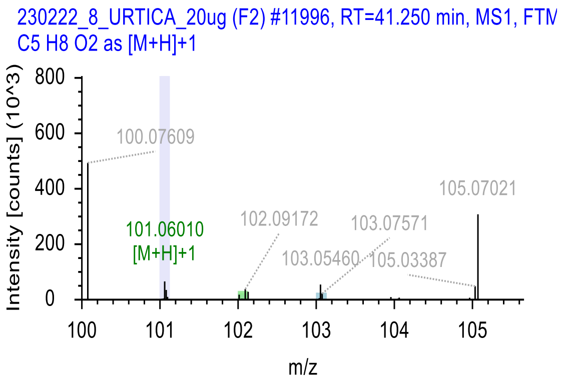

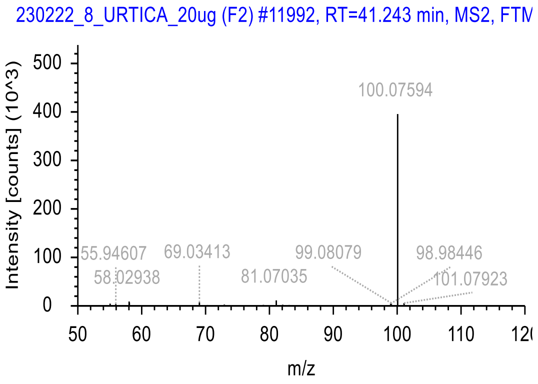

**Compound**	**Molecular formula**	**Mass error (ppm)**	**Annotation MW**	**m/z**	**RT (min)**	**Reference ion**
Azulene	C10 H8	1,02	128,06273	129,07001	32,950	[M+H]+1

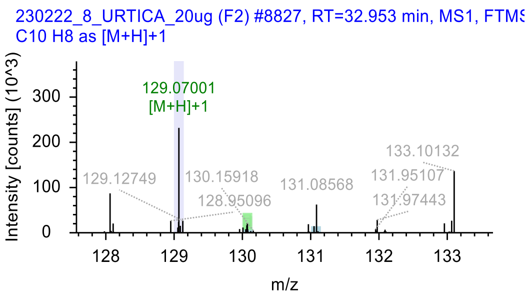

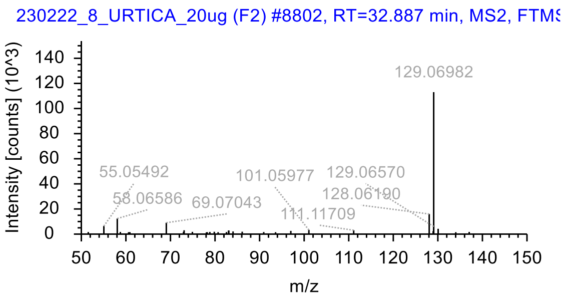

**Compound**	**Molecular formula**	**Mass error (ppm)**	**Annotation MW**	**m/z**	**RT (min)**	**Reference ion**

Bisabolol oxide	C15 H26 O2	-0,49	238,19316	239,20044	35,740	[M+H]+1

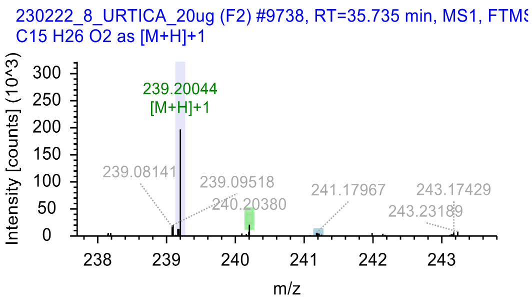

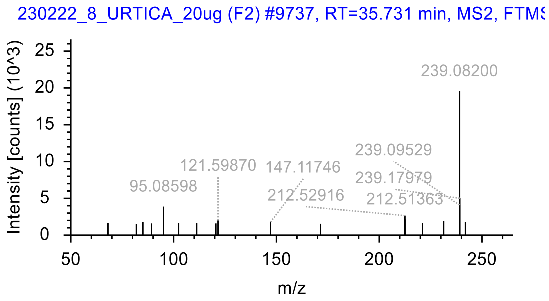

Compound	Molecular formula	Mass error (ppm)	Annotation MW	m/z	RT (min)	Reference ion
Caffeic acid	C9 H8 O4	1,34	180,04250	181,04964	46,627	[M+H]+1

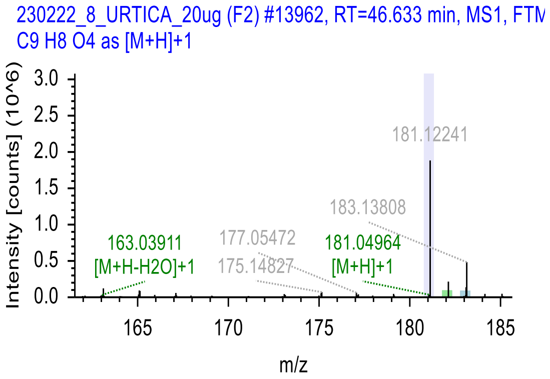

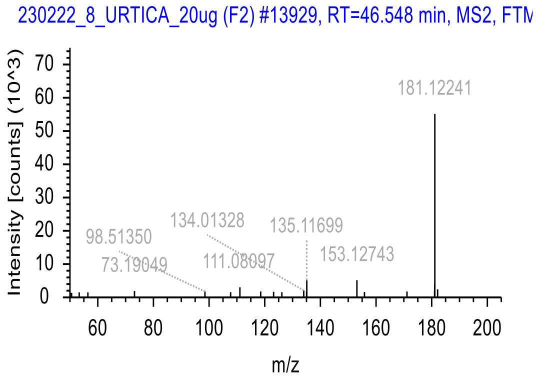

Compound	Molecular formula	Mass error (ppm)	Annotation MW	m/z	RT (min)	Reference ion
Catechol	C6 H6 O2	3,57	110,03717	111,04445	38,796	[M+H]+1

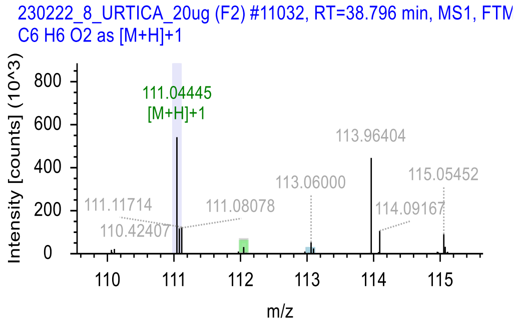

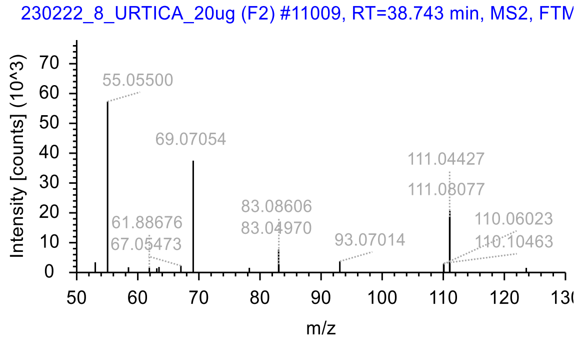

Compound	Molecular formula	Mass error (ppm)	Annotation MW	m/z	RT (min)	Reference ion
Chamazulene	C14 H16	0,16	184,12523	185,13251	42,687	[M+H]+1

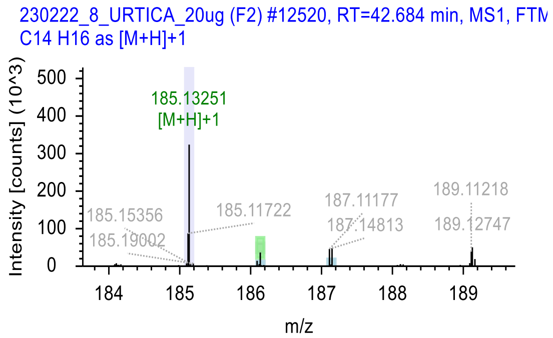

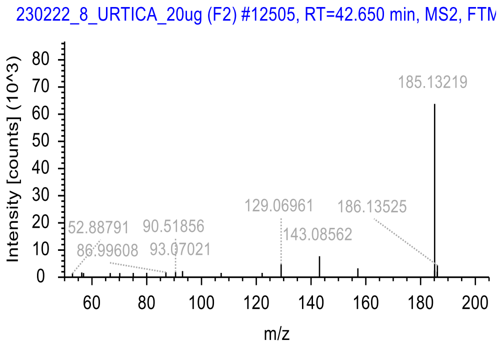

Compound	Molecular formula	Mass error (ppm)	Annotation MW	m/z	RT (min)	Reference ion
Ethyl protocatechuate	C9 H10 O4	0,67	182,05803	183,06531	14,681	[M+H]+1

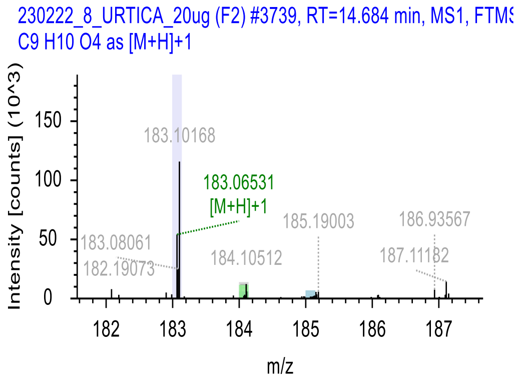

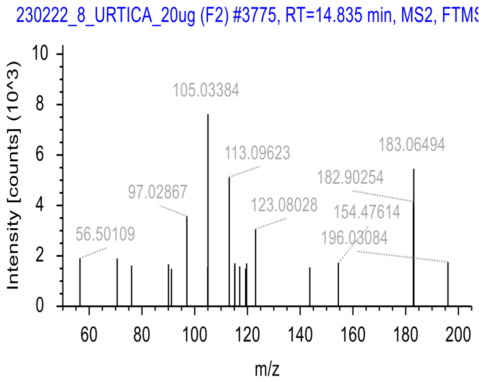

Compound	Molecular formula	Mass error (ppm)	Annotation MW	m/z	RT (min)	Reference ion
p-Coumaric acid	C9 H8 O3	-0,15	164,04732	165,05460	20,856	[M+H]+1

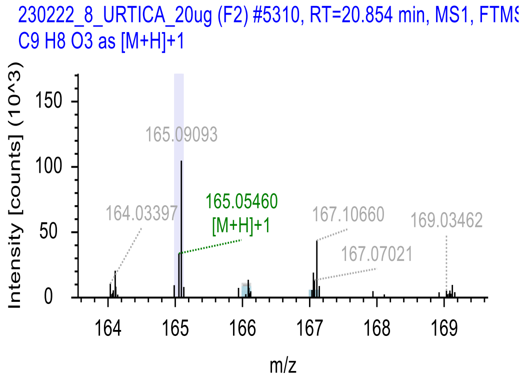

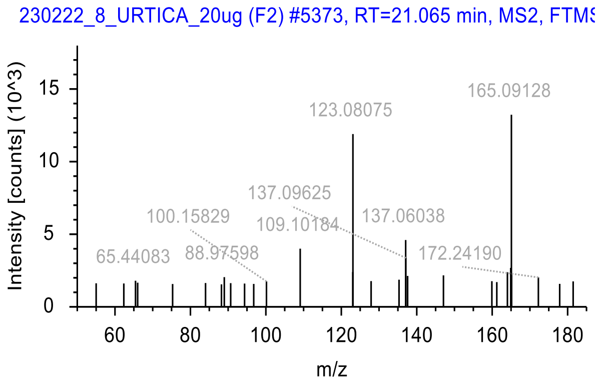

Figure S1. Detailed information related to the identification and confirmation of detected compounds.
